# The Regulatory Role of Signaling Crosstalk in Hypertrophy of MSCs and Human Articular Chondrocytes

**DOI:** 10.3390/ijms160819225

**Published:** 2015-08-14

**Authors:** Leilei Zhong, Xiaobin Huang, Marcel Karperien, Janine N. Post

**Affiliations:** 1 Developmental BioEngineering, MIRA Institute for Biomedical Technology and Technical Medicine, University of Twente, Enschede 7500 AE, The Netherlands; E-Mails: l.zhong@utwente.nl (L.Z.); xiaobinhuang@cqu.edu.cn (X.H.); h.b.j.karperien@utwente.nl (M.K.); 2 School of Life Sciences, Chongqing University, Chongqing 400030, China

**Keywords:** chondrocytes, articular cartilage, signaling, signal crosstalk, hypertrophy, review, osteoarthritis, mesenchymal stem cells, chondrogenesis

## Abstract

Hypertrophic differentiation of chondrocytes is a main barrier in application of mesenchymal stem cells (MSCs) for cartilage repair. In addition, hypertrophy occurs occasionally in osteoarthritis (OA). Here we provide a comprehensive review on recent literature describing signal pathways in the hypertrophy of MSCs-derived *in vitro* differentiated chondrocytes and chondrocytes, with an emphasis on the crosstalk between these pathways. Insight into the exact regulation of hypertrophy by the signaling network is necessary for the efficient application of MSCs for articular cartilage repair and for developing novel strategies for curing OA. We focus on articles describing the role of the main signaling pathways in regulating chondrocyte hypertrophy-like changes. Most studies report hypertrophic differentiation in chondrogenesis of MSCs, in both human OA and experimental OA. Chondrocyte hypertrophy is not under the strict control of a single pathway but appears to be regulated by an intricately regulated network of multiple signaling pathways, such as WNT, Bone morphogenetic protein (BMP)/Transforming growth factor-β (TGFβ), Parathyroid hormone-related peptide (PTHrP), Indian hedgehog (IHH), Fibroblast growth factor (FGF), Insulin like growth factor (IGF) and Hypoxia-inducible factor (HIF). This comprehensive review describes how this intricate signaling network influences tissue-engineering applications of MSCs in articular cartilage (AC) repair, and improves understanding of the disease stages and cellular responses within an OA articular joint.

## 1 Introduction

Osteoarthritis (OA) is a multifactorial complex and chronic disease characterized by progressive degradation of joint cartilage. The underlying molecular mechanisms involved in the pathogenesis and progression of OA are still largely unknown, and currently no disease-modifying therapy is available for OA.

**Figure 1:**
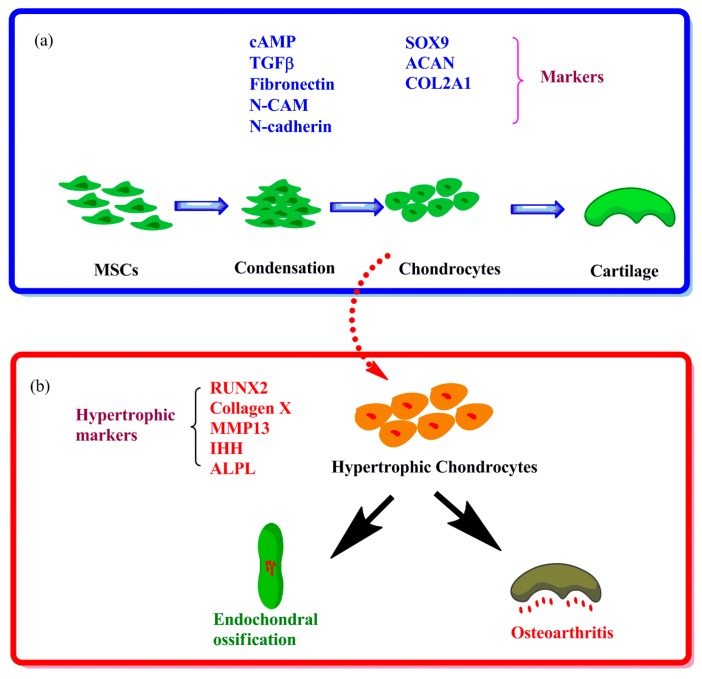
Chondrogenesis of MSCs and hypertrophic differentiation. (**a**) Chondrogenesis is initiated by the condensation of MSCs, and cell-cell contact. The expression of cAMP, TGFβ, Fibronectin, N-CAM and N-cadherin is involved in this process and these factors are necessary for chondrogenic induction, marked by the expression of chondrogenic genes: SOX9, ACAN, COL2A1. Mature chondrocytes begin secreting cartilage matrix primarily consisting of collagen II and GAGs, which are the main components of cartilage; (**b**) Chondrocytes from *in vitro* chondrogenesis of MSCs or *in vivo* cartilage could undergo hypertrophic differentiation, which is characterized by an increase in cell volume and the expression of hypertrophic markers (RUNX2, Collagen X, MMP13, IHH and ALPL). *In vivo*, physiological endochondral ossification and pathological osteoarthritis could be initiated after remodeling, mineralization of the extracellular matrix, and apoptotic death of chondrocytes.

In cell-based cartilage regeneration therapies, the use of mesenchymal stem cells (MSCs) has shown promising results. Evidence showed that MSCs can be differentiated into chondrocytes (marked by Sex determining region Y box 9 (SOX9); Aggrecan (ACAN); Collagen type II (Col2A1)) after a condensation state (marked by Cyclic adenosine monophosphate (cAMP), Transforming growth factor-β (TGFβ), Fibronectin, Neural cell adhesion molecule (N-CAM) and N-cadherin) *in vivo* and *in vitro* [1,2,3] (Figure 1a). However, in the application of human MSCs for cartilage repair *in vivo*, hypertrophic differentiation towards the osteogenic lineage is observed. Prevention of hypertrophy is becoming increasingly important for clinical application of MSCs in cartilage tissue engineering [1,4]. Interestingly, recent data indicate that the healthy chondrocyte phenotype switches toward a hypertrophic phenotype in degenerated cartilage [4,5,6]. Phenomena such as proliferation of chondrocytes, hypertrophic differentiation of chondrocytes, remodeling and mineralization of the extracellular matrix (ECM), invasion of blood vessels and apoptotic death of chondrocytes correspondingly also occur during OA [7]. In addition, transgenic mouse models have shown that deregulated hypertrophic differentiation of articular chondrocytes may be a driving factor in the onset and progression of OA [4]. Therefore, control of hypertrophic differentiation can be exploited as an effective strategy for cartilage repair, and used in bone regeneration, where hypertrophic cartilage could act as a template for endochondral bone formation [1]. However, the exact molecular mechanism underlying hypertrophic differentiation is not understood. Despite numerous studies about the function of single signaling pathways in hypertrophy, studies which explore comprehensive signaling pathways in hypertrophic differentiation of MSCs and chondrocytes have not been published in recent years. Here we discuss how signaling pathways are involved in hypertrophy of MSCs and chondrocytes, how these signaling pathways interplay, and how signal factors changed in OA disease.

### 1.1. Hypertrophy in Chondrogenesis of MSCs in Vitro

MSCs are promising candidate cells for cartilage tissue engineering, as they are present in large quantities in adipose tissue, bone marrow, synovium and cartilage [8] and can be expanded for a number of passages without losing their ability to undergo chondrogenic differentiation. Unfortunately, the phenotype of MSCs in cartilage repair is unstable [9,10]. The expression of cartilage hypertrophy markers (e.g., collagen type X) by MSCs undergoing chondrogenesis, raises concern for a tissue engineering application of MSCs, since chondrocyte hypertrophy in neocartilage could ultimately lead to apoptosis and ossification [11].

### 1.2. Hypertrophy in Articular Chondrocytes during OA Progression

Studies have shown that the development of OA may be caused by activation of hypertrophic differentiation of articular chondrocytes [12]. As Figure 1 shows, during hypertrophic differentiation of chondrocytes in OA, chondrocytes lose the stable phenotype and the expression of Runt-related transcription factor 2 (RUNX2), Collagen type X, Matrix metalloproteinase 13 (MMP13), Indian hedgehog (IHH) and Alkaline phosphatase (ALPL) is detected [13]. Healthy articular cartilage (AC) is a stable tissue that has the potential to resist hypertrophic differentiation and maintain the normal phenotype through an unknown mechanism [14]. The interplay of multiple signaling pathways regulates the fate of chondrocytes, *i.e.*, to remain within cartilage or to undergo hypertrophic differentiation.

**Figure 2:**
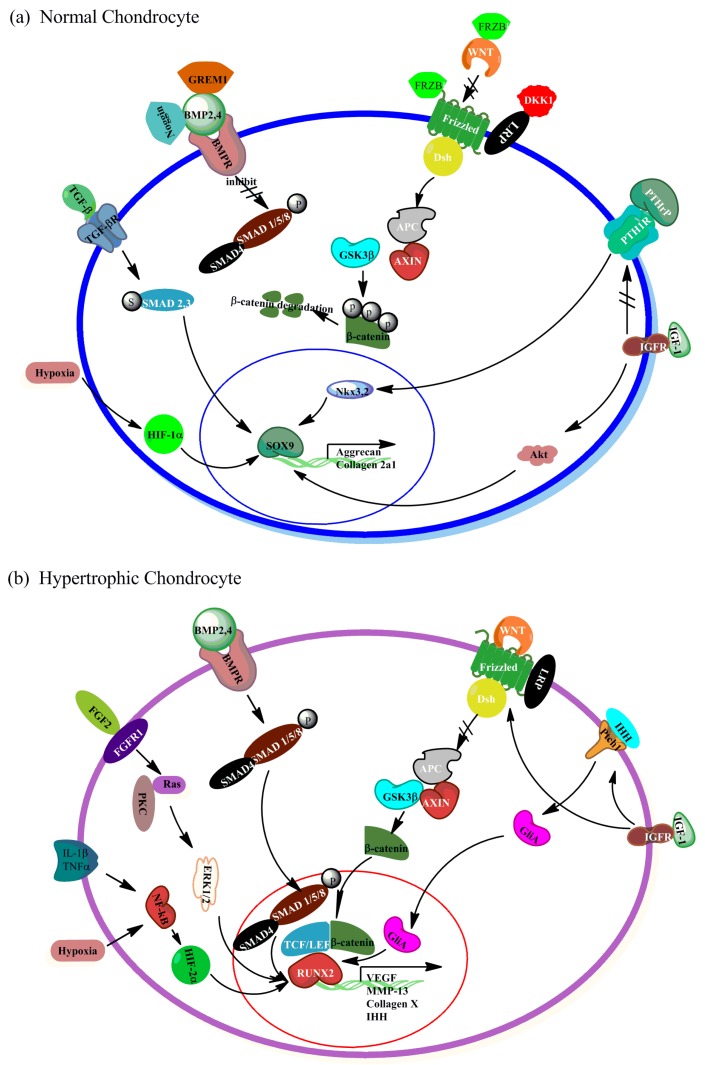
Signal pathways of chondrocyte hypertrophy. (**a**) In normal chondrocytes, signal pathways like WNT, BMP, IHH, *etc.* are regulated by their antagonists (DKK1 and FRZB for WNT, GREM1 for BMP) or other signal factors to get a fine balance to maintain the chondrocyte normal phenotype. The most important transcription factor regulating chondrocytes is SOX9, which is responsible for the expression of main chondrocyte makers including collagen type II and aggrecan. Striked through arrows indicate that the signaling pathway is inhibited by its antagonists; (**b**) In hypertrophic chondrocytes, signal pathways, such as WNT, BMP, IHH, *etc.* are deregulated by their inhibitors or other signal factors, which consequently leads to overexpression of these pathways. Subsequently, the effects of cascade pathways result in activating the transcription factor RUNX2, which regulates the transcription of hypertrophic markers like collagen X, MMP-13, VEGF and IHH.

## 2. Signaling Pathways in Hypertrophy

Multiple signaling pathways have been involved in regulation of hypertrophy-like changes in chondrogenesis of MSCs and chondrocytes. Based on recent literature, the most important related pathways are WNT, Bone morphogenetic protein (BMP)/TGFβ, Parathyroid hormone-related peptide (PTHrP), IHH, Fibroblast growth factor (FGF), Insulin like growth factor (IGF) and Hypoxia-inducible factor (HIF) signaling pathways [15], Figure 2. In each single pathway, several distinct subtypes are involved in the regulation of chondrocyte differentiation and hypertrophy, Table 1.

**Table 1:** The subtypes involved in multiple signal pathways (WNT, BMP/TGFβ, PTHrP, IHH, FGF, IGF and HIF) and their main functions in the regulation of chondrocyte differentiation and hypertrophy.

Signal	Subtypes	Main Functions
WNT	WNT3a	Promotes chondrogenic differentiation; delays chondrocyte hypertrophy
	WNT4	Blocks chondrogenic differentiation; promotes chondrocyte hypertrophy
	WNT5a	Promotes chondrogenic differentiation; delays chondrocyte hypertrophy
	WNT5b	Promotes chondrogenic differentiation; delays chondrocyte hypertrophy
	WNT8	Blocks chondrogenic differentiation; promotes chondrocyte hypertrophy
	WNT9a	Blocks both chondrogenic differentiation and chondrocyte hypertrophy
	WNT11	Promotes chondrogenic differentiation; stimulates RUNX2 and IHH expression
	WNT16	Upregulation is accompanied by the downregulation of FRZB
BMP/TGF-β	BMP2	Induces chondrocyte hypertrophy
	BMP4	Induces chondrocyte hypertrophy
	BMP7	Maintain chondrogenic potential and prevents chondrocyte hypertrophy;
	TGF-β	Promotes chondrogenic differentiation; inhibits chondrocyte hypertrophy
	PTHrP	Blocks hypertrophy by stimulating Nkx3.2 and prevent RUNX2 expression
	IHH	Promotes chondrocyte hypertrophy; Stimulates proliferating chondrocytes to produce PTHrP
FGF	FGF2	Promotes expression of RUNX2
	FGF8	Catabolic mediator with a pathological role in rat and rabbit articular cartilage
	FGF9	Promotes chondrocyte hypertrophy
	FGF18	Promotes chondrocyte proliferation and differentiation in the early stages of cartilage development
IGF	IGF-1	Promotes chondrocyte proliferation and maturation; augments chondrocyte hypertrophy
HIF	HIF-1α	Potentiates BMP2-induced SOX9 expression and cartilage formation, while inhibiting RUNX2 expression and endochondral ossification
	HIF-2α	Increases expression of collagen X, MMP13 and VEGF

### 2.1. WNT Signaling

WNT signaling pathways are highly evolutionarily conserved pathways with crucial roles in embryonic development, patterning, tissue homeostasis, growth, as well as in the onset and progression of a variety of diseases [16]. There are three distinct intracellular signaling cascades well known so far: the canonical WNT/β-catenin pathway, the c-Jun N-terminal kinase (JNK) pathway, and the WNT/Ca2^+^ pathway [17]. The canonical WNT/β-catenin pathway is the most-elucidated pathway, mediated by β-catenin accumulation in nucleus, having strong correlation with chondrocyte hypertrophy. As shown in Figure 2b, in most cases, the presence of WNTs that bind to the WNT receptor Frizzled, results in formation complex of Adenomatous polyposis coli protein (APC), Glycogen synthase kinase 3β (GSK3β) and Axis inhibitor (AXIN), which leads to the release of β-catenin from the complex, followed by β-catenin accumulating in the cytoplasm, and then translocation into the nucleus. There β-catenin forms a complex with T cell-specific factor (TCF)/lymphoid enhancer binding protein (LEF) transcription factors to activate the transcription of target genes [17]. However, in the absence of a WNT ligand, β-catenin is phosphorylated by the destruction complex and subsequently ubiquitinylated and targeted for proteasomal degradation.

Numerous studies have revealed a central role of WNT signaling in cartilage homeostasis. In cartilage, moderate activity of WNT is essential for chondrocyte proliferation and maintenance of their typical characteristics [18], but excessive activity increases chondrocyte hypertrophy and expression of cartilage degrading metalloproteinases [19]. For example, the conditional activation of the β-catenin gene in articular chondrocytes in adult mice leads to premature chondrocyte differentiation with collagen type X expression and the development of an OA-like phenotype [20]. However, ablation of β-catenin in the superficial zone of articular cartilage also strongly increases the expression of aggrecan and collagen type X [18]. SOX9 is the master transcription factor and thus a typical marker of chondrocytes, while RUNX2 usually is expressed highly in hypertrophic chondrocytes. This hypertrophy may be induced by the LEF/TCF/β-catenin complex promoting RUNX2 expression in the redundant WNT signal pathway [21]. Much evidence has shown that the switch between SOX9 and RUNX2 expression determines the progression of mature chondrocytes into hypertrophy in response to canonical WNT signaling [17,22,23,24].

There are several types of WNT ligands, which play different roles in the chondrogenic differentiation and cartilage development. Experiments using retroviral misexpression *in vivo* and overexpression methods *in vitro* suggest distinct roles of different WNTs in the control of chondrogenic differentiation and hypertrophy. WNT4 and WNT8 block chondrogenic differentiation but promote hypertrophy [25,26]. WNT9a blocks both chondrogenic differentiation and hypertrophy [27]. WNT3a and WNT5b promote chondrogenic differentiation but delay hypertrophy [26]. The overexpression of WNT11 in MSCs during chondrogenic differentiation promotes chondrogenesis and stimulates RUNX2 and IHH expression [28]. WNT16 transient expression was found associated with the activation of the canonical WNT pathway, and was present in the early phases of osteoarthritis, its upregulation was accompanied by the downregulation of the secreted WNT inhibitor Frizzled-related protein (FRZB) [29]. WNT5a exhibit dual functions during chondrogenesis of MSCs. At early stages, WNT5a induces chondrogenesis and hypertrophy through intracellular calcium release via G-protein coupled receptor (GPCR) activation [1]. At later stages, it can act as an inhibitor of hypertrophy by activating the phosphoinositide 3-kinase (PI3K)/protein kinase B (PKB or Akt)-dependent pathway, which in-turn activates nuclear factor κ-light chain-enhancer of activated B cells (NF-κB), an inhibitor of RUNX2 [30].

Interestingly, the expression of hypertrophy-related markers in chondrogenesis of MSCs is decreased in the presence of Dickkopf (DKK1), which acts as WNT signaling inhibitor (antagonist) by binding to low density lipoprotein receptor related protein (LRP5/6) through cartilage protective mechanisms [4]. Actually, DKK1, FRZB and Gremlin 1 (GREM1) are regarded as natural brakes on hypertrophic differentiation of articular cartilage [4]. Our studies also found increased hypertrophic differentiation and mineralization and decreased expression of chondrocyte markers in the absence of the WNT inhibitors DKK1 and FRZB during chondrogenesis of hMSCs. In MSC pellet cultures, the inhibition of canonical WNT by DKK1 and FRZB increased the expression of collagen II and aggrecan, but did not affect collagen X expression [25,31,32]. However, the reduction of WNT antagonist secreted frizzled related protein 1 (Sfrp1) in MSCs correlated with an increased amount of cytoplasmic β-catenin and an up-regulation of RUNX2 [33].

### 2.2. Bone Morphogenetic Protein (BMP)/Transforming Growth Factor-β (TGFβ) Signaling

#### 2.2.1. BMP Signaling

BMPs are multi-functional cytokines that belong to the transforming growth factor-β (TGF-β) superfamily. BMP signaling is mediated primarily through the canonical BMP-Smad pathway in chondrocytes [34]. The pathway will be activated when BMPs bind to receptors BMPR-I and II, which phosphorylate Sma and Mad related proteins (Smad) 1, Smad5, and Smad8 (R-Smads). The R-Smads form complexes with Smad4 and translocate into the nucleus, where they bind to regulatory regions of target genes to regulate their expression [35]. BMP has multiple roles during embryonic skeletal development, in addition to mesenchymal condensation and chondrogenic differentiation of MSCs, BMPs induce early cartilage formation [36] and are crucial local factors for chondrocyte proliferation and maturation in endochondral ossification [37,38].

Although BMPs have a protective effect in articular cartilage, they are also involved in chondrocyte hypertrophy and matrix degradation [39,40,41,42]. It was reported that the BMP signaling pathway was primarily activated during fracture healing via endochondral ossification and was detected in hypertrophic chondrocytes [43]. Steinert *et al.* showed that BMP2 and BMP4 induce hypertrophy during the chondrogenic differentiation of human MSC *in vitro* [37]. In another study, BMP2 was found to induce chondrocyte hypertrophy during chondrogenesis of progenitor cells ATDC5, whereas BMP-7 appeared to increase or maintain chondrogenic potential and prevent chondrocyte hypertrophy [44]. *In vivo* studies showed that overexpression of BMP4 in cartilage of transgenic mice resulted in an increased hypertrophic zone, indicating increased differentiation of hypertrophic chondrocytes [45]. As the BMPs also played role in the skeletal development, it may be that BMPs drive the chondrocytes to form bone after ossification, rather than to remain as articular chondrocytes [46]. Therefore, BMPs can be protective for articular cartilage but may have harmful effects on AC by inducing chondrocyte terminal differentiation and contributing to OA progression [31]. Our previous study has shown that addition of GREM1, the inhibitor of BMP signaling was able to slow down the hypertrophic differentiation and decrease the mineralization in the process of chondrogenesis of hMSCs [4]. In addition, another BMP inhibitor, Noggin, can block thyroid-induced hypertrophy by inhibiting BMP4 during MSC chondrogenesis [47].

#### 2.2.2. TGF-β Signaling

TGFβ is a potent inducer of chondrogenesis *in vitro* [48,49]. During chondrogenesis of MSCs, TGFβ is the main initiator of MSC condensation. After aggregation, TGFβ signaling further stimulates chondrocyte proliferation while it inhibits chondrocyte hypertrophy and maturation [50,51,52,53,54,55]. Conversely, the activation of the Smad1/5/8 pathway is able to stimulate hypertrophic differentiation with the consequent expression of the hypertrophic markers collagen X, MMP13 and ALPL during chondrogenesis of MSCs [56]. Although TGFβ is clearly crucial in inhibiting chondrocyte hypertrophy during early phases of mesenchymal condensation and chondrocyte proliferation, its addition to chondrocyte differentiation medium in pellet cultures of MSCs was not sufficient to suppress the onset of hypertrophy [9,10,11].

Most recently, several lines of evidence have suggested that the TGFβ/Smad pathway played a critical role in the regulation of articular chondrocytes hypertrophy and maturation during OA development [57,58,59]. Zuscik and colleagues have shown that treatment of articular chondrocytes with 5-azacytidine (5azaC), an anti-tumor agent that functions by blocking DNA methylation, resulted in a shift of regulatory dominance from maturation suppression via TGFβ signaling to maturation acceleration by BMP-2 signaling, which confirms that a shift in signaling dominance from TGFβ to BMP is sufficient to induce AC maturation [60]. This study also raised the possibility that a similar shift in signaling dominance occurs when these cells progresses inappropriately, such as in osteoarthritis, where the balance between TGFβ and BMP signaling pathways may be broken. It has been suggested that TGFβ inhibits terminal hypertrophic differentiation of chondrocyte and maintains normal articular cartilage through Smad2/3 signals [58,61]. The Smad3 pathway can be activated by TGF-β directly to stabilize the Sox9 transcription complex and inhibits RUNX2 expression through epigenetic regulation [62,63]. Homozygous mutant mice of targeted disruption of Smad3- exon 8 developed degenerative joint disease resembling human OA, characterized by progressive loss of articular cartilage, and abnormally increased numbers of collagen type X expressing chondrocytes in synovial joints [58]. However, TGFβ1 administration has been shown to redirect expanded human articular chondrocytes towards hypertrophy [64]. Moreover, TGFβ can induce synovial lining cells to produce inflammatory factors, such as IL1β and TNFα, which further stimulates articular chondrocyte terminal hypertrophy, depositing collagen type X instead of collagen type II and aggrecan The TGF-β superfamily and its downstream phosphorylation of Smads were reported to exhibit both stimulatory and inhibitory effects on chondrocyte hypertrophy [65].

### 2.3. The Crosstalk between BMP/TGFβ and WNT Signaling in Regulating Hypertrophy

β-catenin crosstalk with TGFβ was reported in hypertrophy regulation in MSCs [66]. In the process of TGFβ-induced chondrogenesis of MSCs, temporal activation of β-catenin led to enhanced chondrogenic induction, further developed into hypertrophy and mineralization phenotype *in vivo*. However, the continuous co-activation of two signaling pathways resulted in hypertrophy inhibition, characterized by the suppressed expression of collagen type X, RUNX2, and ALPL, and did not lead to ossified tissue *in vivo* [66].

It was demonstrated that the crosstalk between WNT and BMP plays key roles in regulating chondrocyte activity in pathogenesis of osteoarthritis, which may be cell type-specific [67]. Papathanasiou and colleagues reported the function and crosstalk between BMP2 and canonical WNT/β-catenin signaling in regulating chondrocyte hypertrophy and matrix metalloproteinase (MMP)/aggrecanolytic ADAMTS (a disintegrin like and metalloproteinase with thrombospondin type I motif) synthesis in OA [68]. In this study, they showed human end-stage OA chondrocytes can produce BMP2 and BMP4. Interestingly, only BMP2, but not BMP4, can drive the expression of low-density lipoprotein receptor 5 (LRP5), which is one of most important co-receptors for WNT signaling that leads to β-catenin stabilization, accumulation, nuclear translocation, and activation of target genes. It can be concluded that the BMP-2-induced WNT/β-catenin signaling pathway activation through LRP-5 induces chondrocyte catabolic action and hypertrophy [68].

This report adds to the accumulating evidence that increased or excessive activation of canonical WNT signaling in chondrocytes is detrimental and contributes to OA cartilage degradation. Recently, studies from our group also indicated that the natural WNT and BMP antagonists DKK1, FRZB and GREM1 inhibit hypertrophic differentiation of hMSCs during chondrogenesis by blocking WNT and BMP pathways [4]. Therefore therapeutic approaches to block or suppress canonical WNT and BMP2 pathways using their natural antagonists may protect cartilage damage in end-stage OA.

### 2.4. Parathyroid Hormone-Related Peptide (PTHrP)/Indian Hedgehog (IHH) Signaling

PTHrP is a member of the parathyroid hormone (PTH) family that blocks hypertrophy by stimulating NK3 homeobox 2 (Nkx3.2) [69] and preventing RUNX2 expression [70]. Huang supposed SOX9 is a target of PTHrP signaling in the growth plate and that the increased activity of SOX9 might mediate the effect of PTHrP in maintaining the cells as non-hypertrophic chondrocytes [71]. IHH is an important factor involved in endochondral ossification and expressed in prehypertrophic chondrocytes [72]. In IHH knockout mice, the proliferation and hypertrophy of chondrocytes are significantly reduced [73]. Evidence has shown that IHH can positively regulate the transcription and expression of collagen type X via Runx2/Smad interactions through downstream transcription factors GLI-Kruppel family members (Gli) 1/2 [74]. Both IHH and PTHrP signaling play crucial roles in regulating the onset of chondrocyte hypertrophy. Vortkamp and colleagues [75] found that IHH stimulated proliferating chondrocytes to produce PTHrP, which in turn accelerated the proliferation of periarticular cells and prevented the onset of chondrocyte hypertrophy, thereby keeping chondrocytes in a proliferating state. This negative feedback loop regulates the balance between proliferation and maturation of chondrocytes, ensuring orderly bone formation [75]. On the other hand, resting chondrocytes at the ends of long bones secrete PTHrP, subsequently suppressing IHH production in the proliferating zone. Chondrocytes outside of this paracrine signaling range produce IHH and undergo hypertrophy [1]. PTHrP forms a feedback loop with IHH to regulate the proliferation and onset of hypertrophic differentiation [76,77,78]. During endochondral bone formation, PTHrP-dependent IHH signaling inhibiting chondrocyte hypertrophy is dominant, thereby obscuring the promoting effect of PTHrP-independent IHH signaling. Other researchers reported that IHH can also function independently of PTHrP to promote chondrocyte hypertrophy [79]. In PTHrP knockout mice, the absence of PTHrP caused diminished chondrocytes and accelerated hypertrophic differentiation, and led to premature mineralization of extracellular matrix and apoptosis [75,80]. However, targeted overexpression of PTHrP under the control of the cartilage-specific collagen type II promoter resulted in the opposite effect of chondrodysplasia through delay of the terminal differentiation of chondrocytes, inhibition of apoptosis and disruption of endochondral ossification [81]. A co-culture model from Jiang and colleagues [82] demonstrated that in healthy articular cartilage PTHrP, secreted by chondrocytes from surface layers, inhibits the hypertrophic potential of chondrocytes residing in the deep layer so as to maintain the homeostasis of articular cartilage, but the effect was not confirmed *in vivo*. In another cell study, it was demonstrated that PTHrP from human articular chondrocytes inhibits hypertrophy of MSCs during chondrogenesis in co-culture, and intermittent supplementation of PTHrP also improves chondrogenesis of MSCs and reduces the hypertrophy [83,84]. A similar phenomenon was observed in MSCs pellet studies, it was shown that PTHrP treatment leads to suppression of hypertrophy but also down-regulates collagen II [49]. However, when cultured under hypertrophy-enhancing conditions, PTHrP could not diminish the induced enhancement of hypertrophy in the MSC pellets [85]. However, other researchers observed a selective hypertrophic inhibition upon PTHrP treatment with stable or even up-regulated expression of collagen II [86,87]. This discrepancy might be linked to the existence of both PTHrP receptor 1 (PTH1R)-dependent and PTH1R-independent pathways [1]. PTH1R knockout mice showed accelerated hypertrophy and were unaffected by treatment with PTHrP, indicating that the inhibition on hypertrophy is dependent on PTH1R receptor binding [88]. The choice of the PTHrP isoform has further been shown to affect the suppressive action on hypertrophy, with isoform 1-34 being the most effective in promotion of chondrogenesis as well as inhibition of hypertrophy [89].

Cell studies have shown that FGF2 combined with PTHrP inhibited the TGFβ responsive COL2A1 and COL10A1 expression and ALPL induction. However, calcification of implanted pellets was not prevented by PTHrP *in vivo* [49]. In another study, the combined delivery of TGF-β3 and PTHrP in nude mice reduced calcification [90]. In addition, the canonical Wnt pathway is known to promote chondrocyte hypertrophy via inhibition of the PTHrP signaling activity [91]. Therefore, PTHrP represses hypertrophic cartilage differentiation whereas WNT and IHH promote hypertrophy of chondrocytes. Hence, the fine balance of the crosstalk between signal pathways is a requirement for the normal phenotype of chondrocytes.

### 2.5. Fibroblast Growth Factor (FGF) Signaling

FGF signaling plays a critical role in controlling chondrocyte differentiation [92]. Specifically, four members of the fibroblast FGF family, FGF2, FGF8, FGF9 and FGF18, have been implicated as contributing factors in cartilage homeostasis [92,93,94,95,96]. FGF2 has been shown to be expressed in proliferating and prehypertrophic chondrocytes, periosteal cells and osteoblasts [97]. In human articular chondrocytes, the binding of FGF2 to FGFR1 activates Ras and Protein kinase C delta (PKCδ), which transfer the signals into the nucleus to positively regulate the expression of RUNX2 by the Raf-MEK1/2-ERK1/2 cascade [98]. Under experimental OA conditions, FGF8 has been identified as a catabolic mediator with a pathological role in rat and rabbit articular cartilage [99]. However, little is known about the precise biological function of FGF8 on human adult articular cartilage. In developing stylopod elements, FGF9 promotes chondrocyte hypertrophy at early stages and regulates vascularization of the growth plate and osteogenesis at later stages of skeletal development. Fgf9^−/−^ mice have normal limb bud development and mesenchymal condensations, but show decreased chondrocyte proliferation in stylopod elements, delayed initiation of chondrocyte hypertrophy and abnormal osteogenesis in skeletal vascularization [95]. In the early stage of cartilage development, FGF18 is expressed in the perichondrium and joint spaces to promote chondrocyte proliferation and differentiation. In Fgf18^−/−^ mice, the phenomenon of delayed mineralization was observed, which was found to be closely associated with delayed initiation of chondrocyte hypertrophy, decreased chondrogenesis proliferation of early stages, delayed skeletal vascularization and delayed osteoclast and osteoblast recruitment to the growth plate [100]. Further studies have shown that FGF18 is necessary to induce VEGF expression by signaling to FGFR 1 and 2 in hypertrophic chondrocytes [100]. The FGF receptor 3 (FGFR3) is a tyrosine kinase receptor, expressed in proliferating chondrocytes and early hypertrophic chondrocytes in the growth plate. Both FGF9 and FGF18 are the major ligands of FGFR3 in the growth plate [101]. Recently, Shung and coworkers found that FGFR3 expression increases the expression of SOX9 and decreases β-catenin levels in cultured mesenchymal cells [102].

The interplay of WNT and FGF signaling is important to determine the fate of MSCs and their subsequent differentiation. FGFR1 appears to act downstream of the β-catenin pathway and serves as a key determinant in the lineage decision of skeletal precursors [103]. Hypertrophic maturation of chondrocytes is highly regulated by the interplay of the FGF, IHH, BMP, and WNT signaling pathways. More specifically, FGF signaling accelerates the speed of terminal hypertrophic differentiation, and acts in an antagonistic relationship with IHH expression [104]. Another study suggests that the FGF and BMP pathways collaborate to promote aspects of hypertrophic chondrocyte maturation [105]. However, cartilage of mice carrying a targeted deletion of Fgfr3 is characterized by increased regions of proliferating and hypertrophic chondrocytes [106]. A study from Weiss and colleagues also showed that FGF2, together with PTHrP, may inhibit chondrocyte hypertrophic differentiation and is therefore necessary to obtain stable chondrocytes [49].

### 2.6. Insulin Like Growth Factor (IGF) Signaling

IGF-1 has been identified as an important growth factor for skeletal development by promoting chondrocyte proliferation and maturation, while inhibiting apoptosis to form bones with appropriate size and strength. IGF-1 transmits signals via the type 1 IGF-1 receptor (IGF1R), which is expressed in the proliferating and prehypertrophic zone chondrocytes of growth plates [107]. Evidence shows that IGF-1 stimulates growth plate chondrocytes at all stages of differentiation [108]. High level of IGF-1 was detected in osteoarthritic human articular cartilage [109]. The local infusion of IGF-1 in rabbit tibial growth plate increased the numbers of both proliferative and hypertrophic chondrocytes and promoted hyperplasia of bony trabeculae within the epiphysis [110]. It has been shown that IGF-1 stimulates the chondrogenic differentiation of MSCs into chondrocytes, and into pre-hypertrophic and hypertrophic chondrocytes [111]. Recombinant adeno-associated virus (rAAV)-mediated IGF-I overexpression delayed terminal differentiation and hypertrophy in the newly formed cartilage, which may be due to contrasting effects upon the osteogenic expression of RUNX2 and β-catenin [112]. Another study demonstrates that IGF-1 enhances chondrocyte hypertrophy by insulin-like actions, and that terminal hypertrophic chondrocytes are reduced in Igf1 null mice [113]. Repudi’s study showed that WNT induced secreted protein 3 (WISP3) inhibits IGF-1 induced collagen X induction, reactive oxygen species (ROS) accumulation and ALPL activity, all of which are associated with the induction of chondrocyte hypertrophy [114]. In addition, Mushtaq also found that IGF-1 stimulated chondrocyte hypertrophy and reversed the growth-inhibitory dexamethasone effects in mouse metatarsal [115]. However, evidence shows that chick embryo chondrocytes maintained their normal phenotype and were prevented to undergo hypertrophic differentiation in the presence of IGF-1 [116]. Clearly, the IGF-I mediated improvement in growth was performed by altering the balance between proliferating and hypertrophic chondrocytes.

IGF-1 signaling also is involved in the interaction between the thyroid hormone and the WNT/β-catenin signaling pathways in regulating growth plate chondrocyte proliferation and differentiation. Evidence showed that IGF-1 and the IGF-1 receptor (IGF1R) stimulate Wnt-4 expression and β-catenin activation in growth plate chondrocytes. Chondrocyte proliferation and terminal differentiation induced by IGF-1/IGF1R can be partially inhibited by the Wnt antagonists FRZB and DKK1 [117]. The IGF-1/IGF1R signaling and IGF-1 dependent PI3K/Akt/GSK-3β signaling can be activated by triiodothyronine (T_3_) in the growth plate, and the chondrocytes undergo proliferation and differentiate to prehypertrophy. It seems that chondrocyte proliferation may be triggered by the IGF-1/IGF1R-mediated PI3K/Akt/GSK3β pathway, while cell hypertrophy is likely due to activation of Wnt/β-catenin signaling, which is at least in part initiated by IGF-1 signaling or the IGF-1-activated PI3K/Akt signaling pathway [117]. The fact that IHH expression was reduced in Igf1^−/−^ mice long bones, whereas expression of PTHrP was increased, suggested that IGF-1 signaling is also required to maintain the IHH-PTHrP loop during skeletogenesis [118].

### 2.7. Hypoxia-Inducible Factor (HIF) Signaling

Healthy articular cartilage is a typical avascular tissue, and chondrocytes are able to survive in low oxygen environments [119]. Hypoxia is considered to be a positive influence on the healthy chondrocyte phenotype and cartilage matrix formation. A recent study from our group has shown that the articular cartilage-enriched gene transcripts of GREM1, FRZB, and DKK1, which are established inhibitors of hypertrophic differentiation, were robustly increased in chondrogenic hMSCs pellets under hypoxic conditions, whereas under normoxia conditions these genes did not increase markedly [120]. Evidence shows that hypoxia enhances chondrogenesis and prevents terminal differentiation through a PI3K/Akt/FoxO dependent anti-apoptotic effect [121]. The hypoxic response is mainly mediated by HIF, which includes three family members, HIF-1α, -2α, -3α [122], particularly HIF-1α and HIF-2α, play an active role in chondrocyte development.

Under hypoxic conditions, the transcription factor HIF-1α accumulates and activates the transcription of genes, which are involved in energy metabolism, angiogenesis, vasomotor control, apoptosis, proliferation, and matrix production. In subcutaneous stem cell implantation studies, HIF-1α was shown to potentiate BMP2-induced SOX9 and cartilage formation, while inhibiting RUNX2 and endochondral ossification during ectopic bone/cartilage formation. In the fetal limb culture, HIF-1α and BMP2 synergistically promoted the expansion of the proliferating chondrocyte zone and inhibited chondrocyte hypertrophy and endochondral ossification [123]. However, HIF-2α, encoded by *Epas1*, was identified as a regulator of endochondral bone formation, and appears to be a central positive regulator of collagen X, MMP13 and VEGF expression by enhancing promoter activities through specific binding to the hypoxia-responsive elements [124]. Inflammatory factors like IL-1β and TNF-α can increase the HIF-2α expression by NF-κB signaling in chondrocytes [124,125]. Further experiments have shown HIF-2α participates in crosstalk with the β-catenin and NF-κB pathways to promote chondrocyte apoptosis and endochondral ossification [126]. RUNX2 and IHH were identified as the possible transcriptional targets of HIF-2α related to endochondral ossification; both of them are involved inthe regulation of hypertrophic differentiation of chondrocytes [124,127]. The gene corresponding to nicotinamide phosphoribosyltransferase (NAMPT) is also a direct target of HIF-2α, and plays an essential catabolic role in OA pathogenesis and acts as a crucial mediator of osteoarthritic cartilage destruction caused by HIF-2α or destabilisation of the medial meniscus (DMM) surgery [128]. There is evidence that HIF-2α causes cartilage destruction by regulating crucial catabolic genes [125] and potentiating Fas-mediated chondrocyte apoptosis [129]. However, Lafont and coworkers found that hypoxia promotes cartilage matrix synthesis specifically through HIF-2α but not HIF-1α mediated SOX9 induction of key cartilage genes [130]. The seemingly conflicting effects of HIF-2α to chondrocyte or cartilage could be induced through different pathways and the differences in experiments performed *in vivo* and *in vitro*, which need to be clarified. The balance between HIF-1α/HIF-2α activities clearly contributes to the control of cartilage homeostasis.

## 3. Conclusions

Chondrocyte differentiation is regulated by multiple signal transduction pathways. Maintaining a normal chondrocyte phenotype and avoiding hypertrophy is important for cartilage repair. SOX9 and RUNX2 are two typical markers in chondrocyte development. SOX9 is expressed in chondrocytes, while RUNX2 is highly expressed in hypertrophic chondrocytes. In most cases, a hypertrophic phenotype was accompanied by high expression of RUNX2 through activation of either of the WNT, BMP, IHH, FGF and HIF signaling pathways. However, TGFβ, IGF-I and PTHrP promote the proliferation of chondrocytes. Here we propose a model in which the balance of these signal pathways adjusts the state of chondrocyte proliferation or hypertrophy through the shifting between SOX9 and RUNX2 transcriptional activities. In the WNT pathway, the LEF/TCF/β-catenin complex can promote RUNX2 expression. BMP/TGF-β signaling has a dual role in the chondrocyte development. TGF-β induces collagen II and SOX9 deposition through Smad2/3 phosphorylation pathway, while BMP2/4 promotes chondrocyte hypertrophy and cartilage mineralization via Smad1/5/8 phosphorylation. PTHrP represses hypertrophic cartilage differentiation whereas IHH signaling positively regulates the hypertrophic phenotype by high transcription and expression of collagen type X and RUNX2. IGF-1 signaling stimulates chondrocyte proliferation by the IGF-1/IGF1R-mediated PI3K/Akt/GSK3β pathway, while cell hypertrophy is likely due to activation of Wnt/β-catenin and IHH signaling by IGF-1. HIF-1α and HIF-2α have a distinct role in the chondrocyte development. The former inhibits the RUNX2 expression, while the latter enhances the expression of collagen X, MMP13 and RUNX2 and promotes the hypertrophic differentiation of chondrocytes. The fine balance of the crosstalk between these signaling pathways is a requirement for normal chondrocyte differentiation and cartilage development.
